# Evaluation of a Virtual Networking Event for Emerging Women Leaders in Global Health

**DOI:** 10.5334/aogh.3728

**Published:** 2022-07-11

**Authors:** Sloka Iyengar, Joanna Ehrlich, Eumihn Chung, Agustina M. Marconi, Aliza R. Karpes Matusevich, Aisha Ahmed Abubakar, Nukhba Zia, Anna Kalbarczyk

**Affiliations:** 1 American Museum of Natural History, New York, NY, US; 2 Texas Department of State Health Services, Houston, Texas, US; 3 Johns Hopkins Center for Global Health, Baltimore, MD, US; 4 University of Madison-Wisconsin, Madison, Wisconsin, US; 5 School of Public Health, University of Texas Health Science Center at Houston, US; 6 Ahmadu Bello University Zaria, NG; 7 Johns Hopkins International Injury Research Unit, Health Systems Program, Department of International Health, Johns Hopkins Bloomberg School of Public Health, Baltimore, MD, US; 8 Johns Hopkins Center for Global Health, Baltimore, MD, US

**Keywords:** Network, global health, women’s leadership, virtual event

## Abstract

**Background:**

Networks are critical for leadership development, but not all networks and networking activities are created equally. Women and people of color face unique challenges accessing networks, many of which were exacerbated during the COVID-19 pandemic. Virtual platforms offer opportunities for global professionals to connect and can be better tailored to meet the needs of different groups. As part of the Consortium of Universities for Global Health annual meeting in 2021, we organized a networking session to provide a networking space for emerging women leaders in global health (i.e. trainees, early career professionals, and/or those transitioning to the field).

**Objectives:**

We evaluated the virtual networking session to better understand participants’ perception of the event and its utility for professional growth and development.

**Methods:**

We distributed online surveys to participants immediately after the event and conducted a 3-month follow-up. Out of 225 participant, 24 responded to both surveys and their data was included in the analysis. We conducted descriptive quantitative analysis for multiple choice and Likert scale items; qualitative data was analyzed for themes.

**Findings:**

Participants represented 8 countries and a range of organizations. Participants appreciated the structure of the networking session; all participants agreed that they met someone from a different country and most indicated they had plans to collaborate with a new connection. When asked if the event strengthened their network and if they will keep in touch with new people, most participants strongly agreed or agreed in both surveys. However, after the follow-up, participants noted challenges in sustaining connections including lack of follow-up and misaligned expectations of networks.

**Conclusions:**

The virtual networking event brought together women in global health from diverse backgrounds. This study found that while networking events can be impactful in enhancing professional networks, ensuring sustained connections remains a challenge. This study also suggests that measures to increase the depth and meaningfulness of these connections in a virtual setting and enabling post-event collaboration can help networks become more inclusive and sustainable.

## Background

While women make up the majority of workers in the health sector globally, this is not reflected in roles of leadership in global health. Women comprise 70% of the healthcare workforce, and 90% of the nursing and midwifery workforce but they hold only 25% of leadership roles. Women in Global Health (WGH) released their annual count of gender parity at the 75th World Health Assembly (May 2022), calculating that only 23% of delegations were headed by women, a 3% decrease from the previous year (2021) [1]. On social media WGH exclaimed that this figure is, “brightly illuminating the long-term trend of women kept out of the decision-making room, a trend that means for every woman’s voice, there are three men making decisions on behalf of women.” Women in global health face specific and unique challenges to reaching leadership roles including lack of mentorship, gender biases, harassment, and gendered networks, institutions, and processes [2,3]. But the documented benefits of gender parity in leadership are emerging- women leaders have been shown to positively impact maternal and health care policies, strengthen health facilities, and reduce health inequalities [4].

Networks are vital instruments in leadership development and are increasingly recognized as essential for career growth. The concept of leadership has also been described as a social network, as leaders participate in and influence the structures of their work and personal social networks [5]. While many traditional leadership programs focus on building knowledge, skills, and attitudes, the social relational process of leadership speaks to the development of leadership networks [6]. Networks provide increased influence and power and access to job opportunities, information, and expertise [7]. When effectively leveraged, networks can propel forward careers.

But not all networks and networking approaches are created equally. Women in varied settings have described perceptions that men’s networks did not fit them due to exclusionary practices, inappropriate networking spaces, inaccessible timing (i.e. after hours), and the incorporation of alcohol or extreme sporting activities [8,9,10]. Women are also more likely to be primary caregivers and less likely to participate in after work activities than men. Restricted access to networks denies women involvement in the exchange and creation of knowledge, resources, and power [11].

During the COVID-19 pandemic, traditional modes of networking largely disappeared, and were replaced by virtual events. But many women have experienced substantial challenges in attending and participating in these events including increased responsibilities at home (i.e. child and elder care), and lack of access to reliable internet connections in low-resource settings [12,13]. There are also documented biases in virtual events for women and minorities who report difficulties speaking up in virtual meetings, being ignored or overlooked by coworkers in virtual calls, and getting interrupted, or ignored [13].

Concurrently, the virtual approach to networking has also created new opportunities for engagement by increasing inclusivity and flexibility [14]. Models that were once Western and male-dominated now have an opportunity to meet professionals where they are, physically (i.e., at home) and mentally. This creates additional opportunities for early-career women who may not otherwise have access to these networks or lack resources for in-person attendance. Virtual spaces also help to address “conference inequity”, a common occurrence in global health, where attendees from low-and middle-income countries (LMICs) are under-represented due to systemic barriers including visa restrictions and high travel costs to venues largely set in Europe and the USA [15].

Cullen-Lester et al. developed a model demonstrating how enhancing social networks, both at the individual and group (team, organizational) level can improve knowledge, skills and abilities, thereby facilitating leadership development [15]. They identify 3 potential pathways to modify how individuals interact: individuals developing competencies; individuals shaping networks, and collectives co-creating networks. The model explains the importance of practice and support in fostering stable social networks as individuals progress to participate in leadership roles and processes and the group coalesces around common goals, expectations, and values.

The annual Consortium of Universities for Global Health (CUGH) [16] meeting is an example of collectives co-creating networks. It is one such space that has historically provided networking opportunities for global health professionals around the world; in 2021 this meeting was held virtually. We sought to leverage this global audience, and the virtual platform, via a 3-hour satellite session held on March 10, 2021, to enhance networking opportunities that focused on emerging women leaders in the field (i.e. trainees, early-career, and/or those transitioning into global health). The event was held in the morning (Eastern Standard Time) to accommodate as many geographic regions as possible. Registration for satellite sessions was free, and not restricted to CUGH attendees only. The event was held entirely in English.

The satellite session was designed to provide a catalytic experience for global health students and junior professionals specifically to explore paths to leadership and non-academic careers in global health and build participants’ networks. The session was divided into 3 parts, 1) a networking activity 2) plenary panel, and 3) participant working groups. Two weeks prior to the satellite session we sent registrants a guide for developing a personal elevator pitch (i.e., a succinct and persuasive introduction) including video examples of model pitches (Supplemental File 1). All participants were asked to come prepared with their own elevator pitches outlining their interests and describing how this is unique or how they might add value to a project, working group, organization, or new job. Participants were randomly assigned breakout rooms and given 30 minutes to give their pitches, and network with each other. We believed that starting the session with a networking activity would establish a collegial and open environment where participants felt actively included.

This paper reports findings from an evaluation of the networking activity to better understand perceptions of the networking event and subsequent utilization of the network for emerging women leaders in global health.

## Methods

### Data Collection

We developed a short post-session survey in Google Forms © that was circulated to all participants via email immediately following the event on March 10, 2021. Two follow-up emails were sent, and data collection ended on March 30.

The survey included a set of 5 demographic questions (i.e., country, student status, degree program, occupation), 3 multiple choice questions, 9 Likert scale questions, and 2 open-ended questions. Likert scale questions (Strongly Agree to Strongly Disagree) asked participants to respond to statements about the connections they made at the event such as “The connections that I made were meaningful” and “I will keep in touch with the new people I met.” The open-ended questions asked participants about other networking activities that have been helpful or interesting and what the value of networks is to them.

Participants who completed the initial evaluation and consented to follow-up were sent another survey via email after 3 months (June 10, 2021). This tool included a subset of Likert scale questions from the first tool for comparison purposes. Participants were also asked the extent to which their new connections further introduced them to others, any types of networking activities or follow-up that occurred, and any outcomes from these activities. Two reminder emails were sent, and data collection ended on June 30. The response rate was 24/225 (10.7%).

### Analysis

Survey data were cleaned and imported to R, a statistical analysis software [17]. Personal identifying variables were excluded, and each respondent was assigned a random ID. The overall dataset was separated into initial survey and follow-up survey: the most complete version of the data set that is used in this analysis is organized by participant ID, based on those who completed both the initial and follow-up survey. The initial data set was further organized such that every response variable was changed from a categorical scale to a numeric scale. The total 14 Likert Scale questions in the survey were revalued such that 1 = “Strongly Disagree,” 2 = “Disagree,” 3 = “Neutral,” 4 = “Agree,” and 5 = “Strongly Agree.” R was also used to generate all of the figures included in this paper.

Descriptive count data was tabulated for each of the demographic questions as well as the Likert-scale questions using the Histogram function of the Data Analysis Add-on in Microsoft Excel ® 2016. Where relevant, comparisons were made between the initial and follow-up surveys and differences in answers from one survey to the next were computed.

We conducted descriptive analysis for the demographic collected variables and the Likert-scale questions. We also compared the answers by the category “US Responder” and “Non-US Responder,” and for the descriptive analysis we assessed frequencies. The Pearson’s chi-squared test was used to calculate differences in the quantitative data as the Likert scale data was categorical. The specified level of significance was a p-value of 0.05. We utilized STATA 16 for the analysis. Incomplete answers were excluded from the analysis.

Qualitative data was analyzed using thematic analysis. Verbatim data from the initial and follow-up survey was downloaded in .xls format. As a first step, the data was explored to identify initial codes emerging from the data. These initial codes were discussed by the team to assess broader themes. Once agreed, the data was transferred to MS Word to further organize based on the broader themes. These resulted in 5 key themes – networking activities, values of networks, connections, outcomes, and expectations. Participants’ quotes were then selected based on the sub-themes within the broader themes.

While quantitative and qualitative data were analyzed separately, the analysis was reviewed and mixed at the data interpretation stage to better understand participant responses.

This research was determined exempt from review by the Johns Hopkins Bloomberg School of Public Health Institutional Review Board.

## Results

Three hundred and fifteen individuals registered for the CUGH satellite session and 225 attended some portion of the session. We were unable to record how many attended the networking event specifically, and while the event called for “global health students and junior professionals,” more mid-career or senior professionals may have attended.

Thirty-five individuals responded to the initial survey; a subset of 24 individuals also responded to the 3-month follow up. For this analysis, we used the complete data set of 24 observations (10.7% response rate), which corresponds to the number of participants who answered both the initial and 3-month follow-up surveys.

### Demographics

Participants represented 8 different countries (4 high-income countries and 6 low-and/or middle-income countries). Twelve participants (50.0%) were from the United States; other participants were from Brazil (n = 2 8.33%), India (n = 2 8.33%), Nigeria (n = 2 8.33%), Australia (n = 1, 4.16%), Germany (n = 1, 4.16%), Ghana (n = 1, 4.16%), Kenya (n = 1, 4.16%), Myanmar (n = 1, 4.16%), and Spain (n = 1, 4.16%).

Ten participants (41.7%) were actively enrolled in an academic program, of whom six (25.0%) were in a doctoral program, three (12.5%) were in master’s program, and one participant (4.17%) was enrolled in a bachelor’s program. Participants were asked to identify their current occupation (participants could choose multiple options); 11 participants (45.8%) reported that they worked at a university, 6 participants (25.0%) were full-time students, 4 participants (16.7%) worked at a nonprofit organization, 4 participants (16.7%) worked in a clinical care setting, 3 participants (12.5%) were self-employed, and 3 participants (12.5%) worked at a governmental organization. One participant (4.17%) reported that they worked as a research assistant and 1 participant (4.17%) volunteered for an academic institution abroad.

When asked why they attended the event (participants could select multiple options), 19 participants (79.2%) reported that they wanted to learn about different careers in global health, 17 (70.8%) wanted to connect with others to increase their network, 16 (66.7%) wanted to learn about women’s leadership, and 6 participants (25.0%) were employed but signed up because they were looking for a new job. Additionally, 1 participant (4.17%) reported that they signed up because they wanted to connect with other women in leadership roles, 1 participant (4.17%) reported that they were unemployed and looking for employment, 1 participant (4.17%) was interested specifically in the discussion around non-academic leadership roles in global health, and 1 participant (4.17%) signed up to support women’s leadership, especially the next generation. Demographic information is displayed in Table 1.

**Table 1:** Demographics of participants that responded to the first survey (column 1) and both surveys (column 2).


VARIABLES	RESPONDENTS TO INITIAL SURVEY (N = 35) N (%)	RESPONDENTS TO BOTH SURVEYS (N = 24) N (%)

*What country are you from?*		

United States	16 (45.71)	12 (50.0)

India	3 (8.57)	2 (8.33)

Bangladesh	2 (5.71)	0.00

Brazil	2 (5.71)	2 (8.33)

Germany	2 (5.71)	1 (4.16)

Ghana	2 (5.71)	1 (4.16)

Nigeria	2 (5.71)	2 (8.33)

Australia	1 (2.86)	1 (4.16)

Burkina Faso	1 (2.86)	0.00

Iran	1 (2.85)	0.00

Kenya	1 (2.86)	1 (4.16)

Myanmar	1 (2.86)	1 (4.16)

Spain	1 (2.86)	1 (4.16)

*Are you currently a student (actively enrolled in an academic program)?*		

No	22 (62.86)	14 (58.3)

Yes	13 (37.14)	10 (41.7)

*If you are a student, what degree program are you in?*		

Bachelor’s	3 (8.57)	1 (4.17)

Master’s	4 (11.43)	3 (12.5)

Doctoral	7 (20.00)	6 (25.0)

Not a student	21 (60.00)	14 (58.3)

*What is your current occupation?*		

I am a full-time student	8 (22.86)	6 (25.0)

I am self-employed	3 (8.57)	3 (12.5)

I work at a governmental organization	3 (8.57)	3 (12.5)

I work at a non-profit organization or NGO	7 (20)	4 (16.7)

I work in a clinical care setting	6 (17.14)	4 (16.7)

I work at a university	16 (45.71)	11 (45.8)

Other	4 (11.43	2 (8.33)


### Networking activities

While the focus of this paper is on the CUGH networking event, participants were asked about other networking activities that have been of help and/or of interest to them. These included a wide range of in-person and virtual networking activities ranging from traditional activities (seminars, conferences, panels, breakout rooms, small group discussions) to contemporary networking activities (speed dating, elevator pitch, social media). A few participants shared.

“This is the first event I’ve been to that specifically focused on networking, and I found the breakout rooms so helpful in meeting new people! I think especially in a virtual conference, the breakout rooms felt more natural and conducive to forming connections with others than trying to direct message people over Zoom or over the conference platform.”

“The little [elevator] pitch was good to get me out of my comfort zone and into networking mode.”

### Value of networks

We asked participants to tell us the value of networks to them. Participants considered networks as a “community” which provides opportunities for professional growth and collaboration with others in the field and allows one to appreciate the diversity inherent in global health. The opportunities ranged from availability of resources to learn, expand current network, and potential new employment options. Networks were described as:

“Invaluable as [they] present numerous opportunities for growth and development.”

“[Networks are] how we get things done! Many hands make light work.”

One participant shared how their initial understanding of the value of networks had changed over time and with experience.

“Early in my career, I didn’t see any value, nor did I understand what [networking] really was. I used to assume that it was a pretentious forced way of showing up and faking interest in a conversation. Luckily, I eventually learned about authentic networking, and it has been significant in advancing my career in the most unexpected ways.”

### New Connections

Participants were asked how many new virtual connections they made, and a connection was defined as an exchange of contact information, a LinkedIn request, or something similar. Twelve respondents (50.0%) after the initial survey and 7 respondents (29.2%) during the follow-up survey indicated that they had made 10 or more connections. Eleven respondents (45.8%) after the initial survey and 15 respondents (62.5%) during the follow-up survey indicated that they had made 7 to 9 connections. No participant in the initial survey and 1 participant (4.17%) in the follow-up survey reported making between 4 and 6 connections. One participant (4.17%) both in the initial and the follow-up survey reported as having made between 1 and 3 connections.

When asked if they had met someone new from a different country after the initial event, all participants either strongly agreed or agreed. After the initial event, participants were asked if they “already had plans to collaborate with someone”; 10 participants (41.6%) were neutral, 10 participants (41.6%)) strongly agreed or agreed, and 4 participants (16.7%) disagreed or strongly disagreed. During both the initial and the follow-up surveys, participants were asked if they would like to collaborate in some way with someone they had met at this event at some point in the future. At the initial survey, 23 participants (95.8%) agreed or strongly agreed and one participant (4.17%) was neutral. At the 3-month follow-up, 21 participants (87.5%) agreed or strongly agreed, 2 participants (8.33%) were neutral, and 1 participant (4.17%) disagreed.

### Further connections

**Figure 1:**
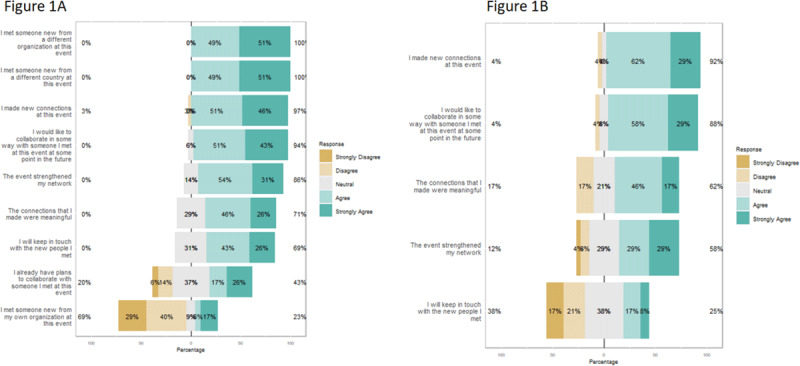
Bar plot summary of Likert scale responses in the inital **(A)** and follow-up **(B)** surveys to the perceived of the networks.

The 3-month follow-up survey asked participants if any of the connections made during the initial event further connected them to members of their network (that is, were they introduced to someone new that was not at the event). Most participants (n = 20, 83.3%) said, “no” such a connection had not taken place, 2 participants (8.33%) made 1 such connection, 1 participant (4.17%) made 3 such connections, and additionally, 1 participant (4.17%) made 5 or more such connections. When asked if respondents had followed up with anyone they met at the event beyond the initial connection (i.e., an informational interview or an email exchange following a LinkedIn connection), 11 individuals (45.8%) indicated that they had not followed up after the initial contact, 10 individuals (41.7%) indicated that they had followed up with their contacts, and 3 individuals (12.5%) were unsure. Given the discrepancy between the number of “new” connections made and attempts to follow-up with them, it is possible that respondents did not understand this question. A breakdown of the number and quality of connections made is presented in Table 2, and a bar plot summary of the Likert scale responses in the initial and the follow-up surveys as to the perceived quality of the networks made by participants is shown in Figure 1.

**Table 2:** Participant motivation to join the networking session (answers to the prompt: “Why did you sign up for this session?”).


VARIABLE	N (%)

*Why did you sign up for this session?*	

I wanted to connect with others to increase my network	17 (70.8)

I wanted to connect with other women in leadership roles	1 (4.17)

I wanted to learn about different careers in global health	19 (79.2)

I wanted to learn about women’s leadership	16 (66.7)

I am employed but looking for a new job	6 (25.0)

I am unemployed and looking for employment	1 (4.17)

I was interested specifically in the discussion around non-academic leadership roles in global health	1 (4.17)

To support women’s leadership, especially the next generation	1 (4.17)


Those who followed-up with new connections used different mechanisms including virtual meetings (via zoom), networking coffee, exchanges via emails, and LinkedIn. The focus of these follow-ups was to identify synergies in participants’ area of work and plan potential collaborations ranging from workshops to opportunities for future work.

“[I conducted] one follow-up zoom call with a connection; it was a good conversation with a number of synergies. I haven’t pursued it further, but we have each-others contact in case of future collaboration opportunities.”

Those who have started collaborations are engaged in manuscript and grant writing and developing training programs.

“We are working together in two groups looking at the data, to identify the ‘nuggets’ that we can develop into a peer reviewed publication. We are writing a grant together and establishing new collaborations.”

### Response changes over time

**Figure 2:**
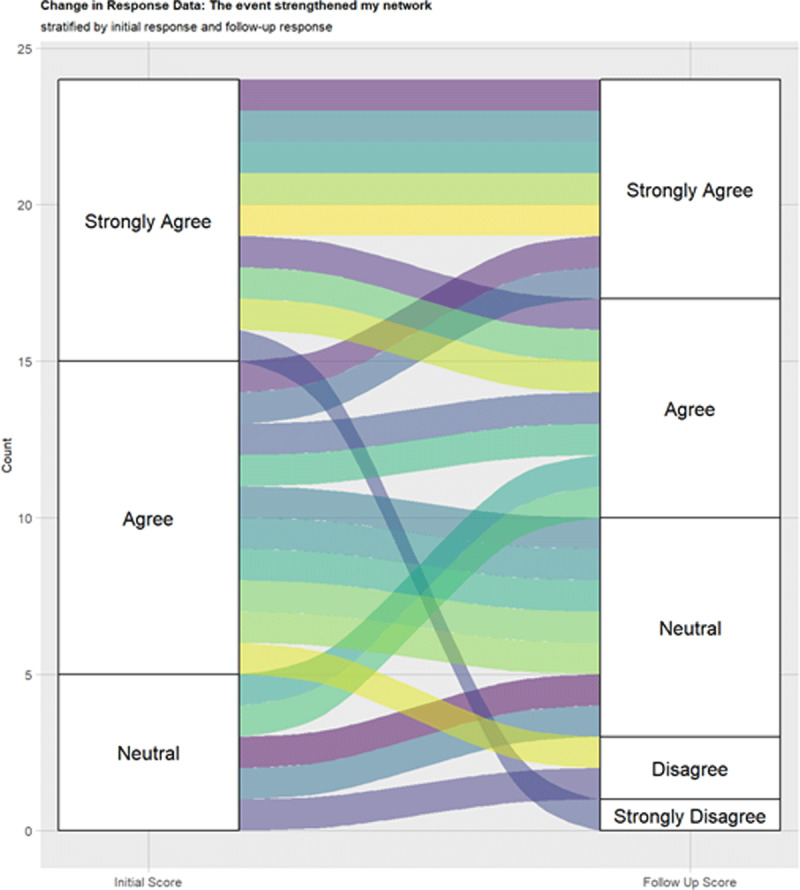
Alluvial plot “The event stregthened my network” during inital and follow-up surveys.

**Figure 3:**
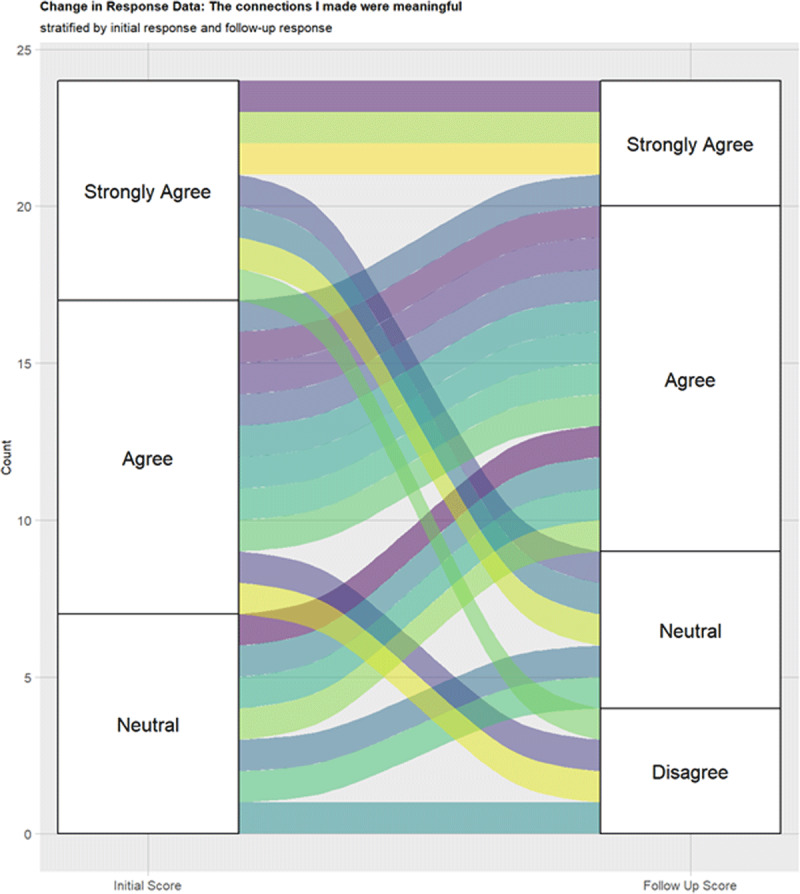
Alluvial plot “The connections I made were meaningful” during initial and follow-up surveys.

Participants were asked a series of Likert scale questions to assess the extent to which they agreed with statements about their new connections because of the event. To the prompt “the event strengthened my network,” analysis showed no changes in responses between the 2 surveys. The alluvial plot (Figure 2) displays participant responses with 1 respondent going from “strongly agree” to “strongly disagree,” and 1 respondent going from “agree” to “strongly disagree.” To the prompt “the connections I made were meaningful,” the alluvial plot (Figure 3) shows that there was no significant change between the surveys, with 1 respondent going from “strongly agree” to “disagree,” and 2 respondents going from “agree” to “disagree”.

**Figure 4:**
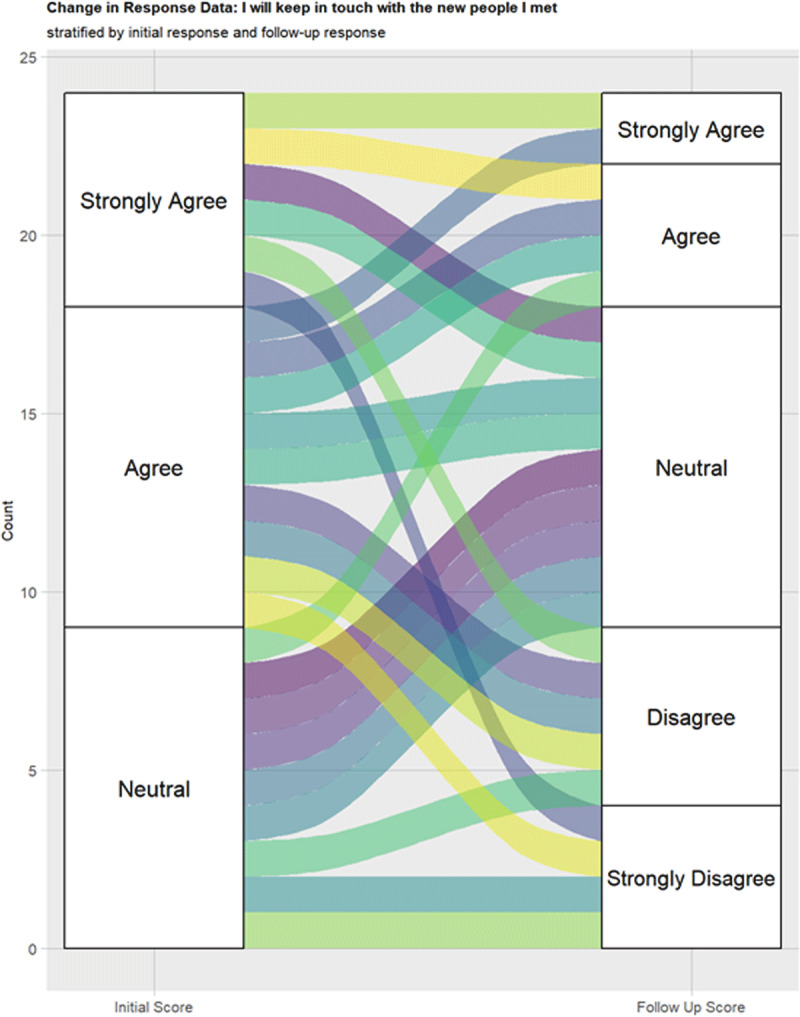
Alluvial plot “I will keep in touch with the new people I met” during inital and follow-up surveys.

When asked if participants “will keep in touch with the new people they met,” 15 participants (62.5%) agreed or strongly agreed, 9 participants (37.5%) were neutral and none of the participants disagreed during the initial survey. However, during the follow-up survey, 9 participants (37.5%) were neutral, 9 (37.5%) disagreed or strongly disagreed, and 6 (25.0%) agreed or strongly agreed. The alluvial plot (Figure 4) displays changes in responses over time.

### Expectations of networks

The follow-up survey explored expectations participants had from networks. These varied based on participants and their background. For students, networks were viewed as an opportunity to assess various career paths, and to find mentors.

“I love meeting new people, making genuine connections, and learning about the different career paths that others are on. I’m currently a graduate student, and am very undecided about what I want to do after my PhD, so I really enjoy networking to gain new insights and perspectives about what careers are possible…”

While for mid-level professionals, the expectation is to connect with other professionals in the field and mentees.

“I consider my career to be at mid-level. So, I’m both looking to be both a mentee and a mentor. The missing key in my career as I ‘move up the ladder’ is how to manage the fine line of being perceived as ‘aggressive’ when there is a need to be ‘assertive.’ So, a survey sent out to everyone to understand their specific needs and a trying to pair up individuals (even if one pairing per person), would be amazing. And more routine/regular events so everyone gets used to the names and faces and personalities. It’s hard to engage connections based on one encounter (virtual or otherwise).”

The participants valued shared experiences and learning from others in the field about their successes and failures. Some areas of focus included building leadership skills, mentorship, and cultural understanding.

“Connecting with others to learn from their experiences (success and failure) and to be able to apply new methods to our work. Would also be interested in partnering on a project.”

The participants did note that while the virtual event provides opportunity to make an initial contact, the long-term and sustainable nature of potential collaboration is not clear.

“…In general, I enjoyed the Women Leaders in Global Health event because it made networking feel less transactional than the word connotes. However, I think that due to the virtual format, a lot of the more organic ways of connecting with other people were extremely difficult to achieve. So, although I met a lot of interesting and inspiring women, I’m not sure whether those connections were and/or are sustainable past the initial meeting. Maybe follow-up is a skill that I need to work on?”

To enhance the networking experience, participants shared the need for more opportunities to connect with professionals in their field and country through events such as casual conversations, workshops, conferences, and professional development events.

Participants suggested different ways to support connection building. These include providing templates for conversation starters, using a matching process to pair members of a network, a searchable database consisting of roles and career information on members of a network, sharing opportunities for collaborations and employment.

“I do part-time global health work so only need to lean into networking from time-to-time at transition points in projects, etc. – would be nice to have something that is easy to go back to besides form some hastily written notes and jotted down email addresses/LinkedIn profiles!”

## Discussion

Developing effective networking opportunities for women can help to facilitate strong professional connections and influence their trajectory towards leadership roles. Given the inherent challenges of networking, perhaps even more so in the virtual setting [18], data from this study can provide a better understanding of successes and opportunities of virtual networking.

The data from the CUGH survey shows that the virtual networking event enabled new, global connections, with all respondents answering favorably when asked if they met someone new from a different country. The opportunities that virtual networking present are of particular importance to women in global health, for whom the barriers to in-person networking are immense. Strengthening virtual networking opportunities can increase access to networking and inclusivity of networks for women global health professionals around the world [19].

The data also shows that the CUGH satellite session facilitated the opportunity to expand the participant’s networks, and that most respondents initially felt the connections they made were meaningful. Over 90% of survey respondents indicated that they made at least 7 connections at the event. The sheer number of virtual connections established at the event points to the great potential for virtual networking to quickly expand networks. But, the effectiveness of virtual networking often relies on continued interaction and collaboration to strengthen the connections made and increase each participant’s perceived value of the network [20]. Survey responses suggest that participants may have had differing expectations for the networking event itself or the types of connections that they would make at the event, which led to diminishing responses over time.

Clearly defining the goals of networking events and communicating participant expectations has the potential to influence participants’ perceptions of the event and may encourage more meaningful connections and follow-through when participant expectations are closely aligned with those of the organizer. Understanding members’ motivation for joining a network and participating in activities is also important to tailor events and activities to meet members’ needs. In general, motivations for networking include building social capital [21], enhancing knowledge and learning, promoting skills development, and to facilitate career success [22]. People are more likely to become deeply engaged with a network that aligns with their expectations and provides tangible resources to help them reach their goals.

Data from the CUGH satellite session point to potential discrepancies among participants’ expectations, their perceived value of the network, and intent to collaborate after the event. In general, more favorable responses were recorded during the initial survey compared to the follow-up survey that was conducted 3 months later. Despite most respondents indicating that many new connections were made, there was an 18.4% reduction in the participants who stated that they made meaningful connections at the event, and a 20.8% reduction in those who intended to keep in touch with their connections after the follow up survey. In addition to differing expectations, diminishing enthusiasm, and barriers to maintaining the connections over time may have contributed to the respondents’ perception of the event over time.

Creating long-lasting and meaningful connections through virtual networking may pose a challenge compared to traditional in-person networking. As one participant in the CUGH satellite session noted, establishing strong connections was more difficult through the virtual platform. Direct human and social interaction have long been an important element of networking. Data from past networking studies show that early career women prefer organic networking, which relies on building connections based upon shared interests. With the shift to virtual networking, recreating these experiences can be a challenge [23]. Designing purposeful and interactive activities to facilitate active participation and discussion, and peer-to-peer interaction is important when planning virtual networking events [24]. Additionally, the use of the chat function in online meeting platforms can help democratize opportunities for speaking up [25], which has historically been difficult for women to do [13]. Maximizing the sociability of virtual networking events can bring elements of traditional in-person networking to the virtual space. One way to foster longevity of networks is enabling opportunities for knowledge sharing, help in defining and clarifying career goals, provide social support, decrease isolation, and strengthen networks through interconnections [26].

### Recommendations

Virtual networking has the potential to grow women’s global health networks and expand their reach, but challenges persist that threaten the depth and meaningfulness of connections that may not exist when networking in-person. We offer 4 recommendations to strengthen virtual networks for women in global health.

### Recommendation 1

Facilitate breakout sessions or networking activities that promote peer-to-peer interaction. Establishing organic connections based on participants’ shared interests can be more difficult through virtual networking. Virtual events typically rely on the chat or Q&A function to facilitate communication between participants and presenters, whereas in-person networking provides ample opportunity for discussion and socializing with other attendees. The CUGH satellite session provided a structured, intentional networking activity that participants indicated helped to develop many meaningful connections. Including purposefully designed, interactive activities to improve the socialization of the event can help to bring some of the benefits of in-person networking to the virtual space.

### Recommendation 2

Networks should work towards aligning expectations of members with the events and activities planned within the organization. Understanding members’ expectations is essential to align participants’ perceptions of the events and activities and try to organize activities to meet the needs of the participating members. This may be facilitated by circulating materials ahead of time such as the agenda and instructions for event activities (i.e. “how-to” videos on the elevator pitch).

### Recommendation 3

Facilitate platforms for post-event communication and collaboration. The inherent challenges and limitations of virtual networking can make developing deep connections more difficult. Therefore, follow-up and continued communication and collaboration after virtual events may be lacking. Event organizers could schedule follow-ups which would save a date on people’s calendars, rather than individuals making it themselves. Participant emails could be collated, with permission, and shared after the event to facilitate connections. Networks that engage virtually should place importance on creating and utilizing platforms that provide opportunities for those seeking employment, collaboration, or to provide social cohesion in a virtual world. Networks can distribute information about upcoming events and network updates through newsletters or social media platforms to keep members engaged. After the CUGH satellite session, attendees were encouraged to join the “Emerging Women Leaders in Global Health (EDGE)” Slack channel to facilitate continued interaction among new connections and incorporate new members more deeply into the network. The intentional use of communication platforms and tools, such as Slack, WhatsApp, or LinkedIn, may be helpful in supporting network activities, and helping members fully understand the network’s potential and how it can be leveraged to support their goals.

### Recommendation 4

Make virtual networking events more approachable and tangible, creating something for everyone and improving participants’ perceptions of the network. In a global network, this can be accomplished by organizing events at times so that people from multiple time zones can join, holding multiple sessions to accommodate those all over the world, and providing translation facilities in the participants’ local language. While our event used text chat functions in addition to voice, sign language interpreters could also increase accessibility.

While these recommendations can positively influence the creation and sustainability of women’s global health leadership networks, actualizing them entails both financial and time investment. Additionally, a strong, sustained commitment to creating and sustaining networks of women leaders is essential.

### Strengths and limitations

The pandemic has revealed the power and promise of technology [27], and this study provides unique insights on navigating a virtual networking world, specifically for women in global health. The authors represent a subset of participants of the CUGH satellite session which ensures the insights and perspectives are representative of the session participants, and not just the session organizers.

This study was limited in its sample size due to a low response rate of the post-event survey. The small sample reduces the generalizability of these results. Future work on this topic would benefit from an increased sample size, which can be achieved by providing creative incentives for participants that finish both surveys, and by announcing and advertising the study and survey as part of the event. While this study does not have the ability to assess differences in engagement and participation as a function of participant demographics and other characteristics, future work would benefit from such detailed analysis.

Another limitation was the subset of participants that did not respond to the second survey. While the small sample size precludes a sensitivity analysis, we can hypothesize that the reason behind the non-responsiveness to the second survey may also be challenges in sustaining meaningful connections over time.

Additionally, since the follow-up survey was conducted 3 months after the virtual event, the responses may have been impacted by some recall bias.

## Conclusion

Virtual networking has the potential to grow networks and expand their global reach. A purposefully designed, interactive virtual networking event can increase access to networking and improve the inclusivity of diverse women in networks around the world. But challenges persist that threaten the depth and meaningfulness of connections that may not exist when networking in-person. Virtual networks should try to plan events and activities that maximize sociability and peer-to-peer interaction to facilitate organic connections based on shared interests. Networks should also place emphasis on aligning expectations between members and organizers, facilitating post-event collaboration, deeply incorporating members into the network, and making networking events approachable and tangible for all participants.

## Additional File

The additional file for this article can be found as follows:

10.5334/aogh.3728.s1 Supplemental File 1. Elevator Pitch Development Guide.
