# Supplementary material for: Evaluation of a Virtual Networking Event for Emerging Women Leaders in Global Health
Source: Ann Glob Health. 2022 Jul 11;88(1):54. doi: 10.5334/aogh.3728 (PMC9284988; doi:10.5334/aogh.3728)
Supplement: Supplemental File 1. — Elevator Pitch Development Guide. [file agh-88-1-3728-s1.pdf]

Global Speed Networking for Emerging Women Leaders Resource Library

Focus: Bumper stickers and Beyond

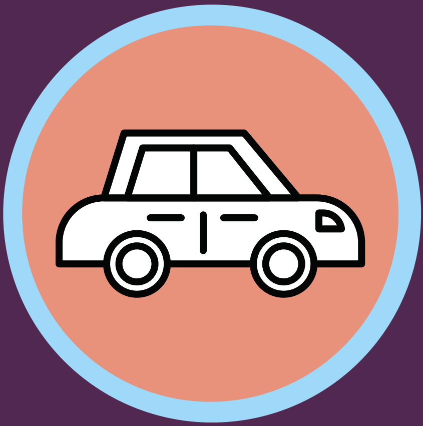

University of Minnesota SPH
"Elevator Pitches and Bumper Stickers"

The elevator pitch is a formal introduction that focuses on your education and professional development. Alternatively, your “bumper sticker” weaves in a personal tagline, uses short and focused statements, and is more conversational than an elevator pitch. “Bumper stickers” are organized by your responses to questions such as “I really enjoy working in/on ____”, “I have focused in/on _____,” and “I am interested in/on ___.”

Successful networking doesn't stop with the event! Consider arranging private networking events with 5-10 people you’d like to connect with on a deeper level or have similar interests in. By taking the initiative of setting up an e-mail chain or Zoom meeting, you are actively opening yourself and others to "accidental bumps" in our network!

Harvard Business Review
"How to Network when there are no networking events"

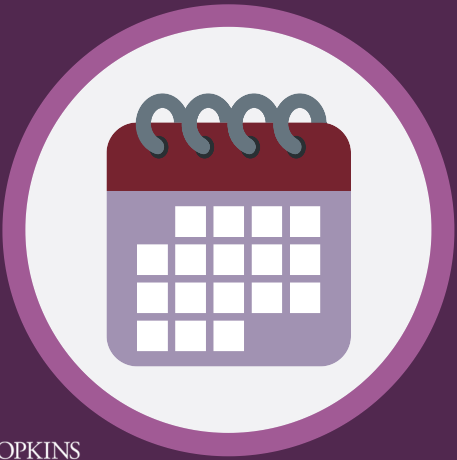

JOHNS HOPKINS
CENTER *for* GLOBAL HEALTH

Global Speed Networking for Emerging Women Leaders Resource Library

Focus: Bumper Stickers and Beyond

We hope that the resource guides over the past few weeks have been insightful and helpful in preparing for the event Wednesday morning! As a reminder, the typical format for speed networking is called a round robin.

How the day will work:

- When the doors open, participants can enter the Gather space early to get to know the platform. Please be sure to sign in at the white Welcome Table.
- A bell will ring to indicate the start and end of each round (5 minutes)
    - Introduce yourself to the participant sitting across from you and share your purpose for being there, and then switch. (Before the event, please practice a 30-second statement that includes your name, title or occupation, and what you are looking for at the event (i.e. a job, a connection, collaboration). Then spend the remaining time getting to know each other.
    - Be sure to exchange contact information such as email address or your LinkedIN profile. One suggestion is to organize your contact information beforehand, almost like a "virtual business card" that is ready to be "copied and pasted" into the chat box.
- At 9:15 am, participants will be able to mingle openly around the Gather space for the rest of the event.

Practicing your Elevator Pitch and Bumber Sticker

The University of Minnesota School of Public Health has a resource page that discusses the "Elevator Pitch" and your "Bumper Sticker". The elevator pitch is a formal introduction that focuses on your education and professional development. Alternatively, your "bumper sticker" weaves in a personal tagline, uses short and focused statements, and is more conversational than an elevator pitch.

"Bumper stickers" complete the following statements

- "I really enjoy working in/on ____"
- "I have focused in/on _____"
- "I am interested in/on ___"
- "Some of my current projects include ___"

Although you may not be able to present everything you would like to say, you can always continue conversations outside of the event! In this Harvard Business Review article, participants are encouraged to put together their own private networking events with 5-10 people you'd like to connect with or have similar interests in. By taking initiative to set up an e-mail thread or a Zoom meeting, you are directly placing yourself and others involved in the path to new opportunities! Even if you don't find a point of potential collaboration or connection with someone you met, think of who else in your network you know who might want to meet that person and make the connection for them!
